# Correction: One-pot aqueous synthesis of highly strained CdTe/CdS/ZnS nanocrystals and their interactions with cells

**DOI:** 10.1039/d5ra90131f

**Published:** 2025-12-01

**Authors:** Mehriban Akin, Johanna-Gabriela Walter, Antonina Lavrentieva, Imme Kretschmer, Lydia Sandiford, Alix Le Marois, Rebecca Bongartz, Pooyan Aliuos, Klaus Suhling, Frank Stahl, Mark Green, Thomas Scheper

**Affiliations:** a Gottfried Wilhelm Leibniz University of Hannover, Institute of Technical Chemistry 30167 Hanover Germany scheper@iftc.uni-hannover.de; b King's College London, Department of Physics The Strand WC2R 2LS London UK mark.a.green@kcl.ac.uk; c Hannover Medical School, Biomaterial Engineering 30625 Hannover Germany

## Abstract

Correction for “One-pot aqueous synthesis of highly strained CdTe/CdS/ZnS nanocrystals and their interactions with cells” by Mehriban Akin *et al.*, *RSC Adv.*, 2015, **5**, 7485–7494, https://doi.org/10.1039/C4RA13386B.

Mehriban Ulusoy was listed as one of the authors in the above-mentioned published article. This is to inform the readers that their name has changed to Mehriban Akin and their name has been updated in the published article.

